# Development and validation of a succinylation-related prognostic model for esophageal squamous cell carcinoma based on multi-omics bioinformatics analysis

**DOI:** 10.1186/s41065-026-00653-2

**Published:** 2026-02-17

**Authors:** Beibei Hua, Weiwei Wang, Huijie Huang, Xuezhi Chang, Tao Ye

**Affiliations:** 1 Department of Radiation Oncology, The Friendship Hospital of Ili Kazakh Autonomous Prefecture, Yining, Xinjiang China; 2 State Key Lab of Pathogenesis, Prevention and Treatment of High Incidence Diseases in Central Asia, Xinjiang Medical University, Urumqi, China; 3 Department of Pathology, The Friendship Hospital of Ili Kazakh Autonomous Prefecture, Yining, Xinjiang China; 4 Department of Thoracic Surgery, The Friendship Hospital of Ili Kazakh Autonomous Prefecture, No. 92, Stalin Street, Yining City, Xinjiang Uygur Autonomous Region 835000 China; 5 Ili & Jiangsu Joint Institute of Health, Yining , Xinjiang China

**Keywords:** ESCC, Succinylation, Prognostic signature, TME, Bioinformatics

## Abstract

**Background:**

Esophageal squamous cell carcinoma (ESCC), an aggressive malignancy with poor prognosis, requires reliable prognostic biomarkers. Protein succinylation, a critical post-translational modification implicated in cancer biology, has not yet been systematically investigated for its prognostic significance in ESCC.

**Methods:**

Transcriptomic data from GSE53624 (*n* = 119) and The Cancer Genome Atlas (TCGA)-ESCC (*n* = 95) cohorts were analyzed in this study. Succinylation-related genes (SRGs) were identified via weighted gene co-expression network analysis (WGCNA), a curated SRG set, and differential expression analysis. A prognostic signature was constructed using LASSO Cox regression and validated internally and externally. Immune infiltration was assessed by CIBERSORT/ssGSEA. miRNA-mRNA and protein-protein interactions (PPIs) and drug sensitivity were evaluated. Gene localization was confirmed with single-cell RNA sequencing (scRNA-seq).

**Results:**

41 high-confidence SRGs associated with ESCC were identified. A robust seven-gene prognostic signature (IGFBP3, CMA1, FN1, CTSG, TIMP1, MBL2, and SP5) was established. Patients stratified into high- and low-risk groups exhibited significantly different overall survival (OS) in both the training cohort (GSE53624, *P* < 0.001) and the validation cohort (TCGA, *P* = 0.026). The risk score remained an independent prognostic factor and was incorporated into a predictive nomogram. High-risk tumors were characterized by an immunosuppressive tumor microenvironment (TME), with reduced eosinophil and natural killer (NK) cell infiltration and increased monocyte abundance. In addition, signature genes were associated with resistance to multiple anticancer agents. scRNA-seq analysis revealed predominant expression of these genes in malignant cells, fibroblasts, and myeloid cells.

**Conclusion:**

A novel seven-gene succinylation-related signature was established and validated as an independent prognostic biomarker for ESCC. This signature captures immunosuppressive features of high-risk tumors and may provide a foundation for individualized therapeutic strategies.

**Supplementary Information:**

The online version contains supplementary material available at 10.1186/s41065-026-00653-2.

## Introduction

Esophageal squamous cell carcinoma (ESCC), a primary reason behind cancer-related mortality globally, features clinically aggressive behavior and unfavorable prognosis [1, 2]. Its pronounced heterogeneity highlights the urgent need to identify robust prognostic biomarkers and elucidate the molecular mechanisms underlying tumor progression, thereby enabling personalized therapeutic strategies [3]. Post-translational modifications (PTMs), which play critical roles in regulating protein function, have increasingly been recognized as key contributors to tumorigenesis [4, 5]. Among these, succinylation [6, 7], a relatively recently discovered PTM, has emerged as a key regulator of diverse cellular processes, including metabolism, inflammation, and gene expression [8, 9]. Despite its potential significance, the roles of succinylation and succinylation-related genes (SRGs) in ESCC pathogenesis and progression remain unexplored [10, 11].

High-throughput transcriptomic technologies, including bulk RNA sequencing and single-cell RNA sequencing (scRNA-seq), have enabled unprecedented systematic characterization of the complex molecular landscapes of cancer [12, 13]. Integration of multi-omic datasets facilitates the identification of functionally relevant gene modules and the construction of molecular signatures with potential clinical utility. In addition, characterization of the tumor immune microenvironment (TIME) is essential, as immune cell infiltration (ICI) profoundly influences both prognosis and therapeutic response [14].

It is hypothesized that SRGs are pivotal in ESCC pathogenesis and that an SRG-derived gene signature could be a robust prognostic tool while providing insight into underlying biological mechanisms. To evaluate this hypothesis, transcriptomic data from multiple public cohorts were integrated. A core set of succinylation-related hub genes in ESCC was identified through differential expression analysis, a curated SRG list, and weighted gene co-expression network analysis (WGCNA). Based on this core gene set, a novel seven-gene prognostic signature was constructed and rigorously validated using LASSO Cox regression. Subsequently, a prognostic nomogram incorporating clinical parameters was established; differences in the immune microenvironment between high- and low-risk groups were characterized; upstream regulatory networks involving miRNAs and transcription factors were explored; potential drug sensitivities were predicted; and the cellular specificity of signature genes within the ESCC tumor microenvironment (TME) was validated using scRNA-seq data. This comprehensive multi-omic analysis establishes a succinylation-based biomarker for risk stratification and provides novel insights into the molecular mechanisms underlying ESCC.

## Materials and methods

### Data acquisition and preprocessing

Bulk RNA-sequencing and pertinent clinical information for ESCC were from two independent public cohorts. The GSE53624 dataset was from the Gene Expression Omnibus (GEO, https://www.ncbi.nlm.nih.gov/geo/) via GEOquery in R v2.76.0, comprising 119 paired ESCC and adjacent normal tissue samples. This cohort was randomly divided into a training set (*n* = 60) and an internal test set (*n* = 59) in a 1:1 ratio for model development and initial validation. For external validation, RNA-seq data (FPKM format) and corresponding clinical information for 95 patients with ESCC were retrieved from The Cancer Genome Atlas (TCGA) (https://portal.gdc.cancer.gov/), forming an independent external validation cohort (*n* = 95). To investigate the cellular specificity of the identified genes, single-cell RNA-sequencing (scRNA-seq) data were obtained from the GSE160269 dataset generated using the 10X Genomics platform, comprising 56,179 high-quality cells derived from ESCC tissues. A curated list of SRGs was obtained from GeneCards (https://www.genecards.org/) using the keyword “succinylation” and was used for subsequent analyses.

### Differential gene expression analysis

For the GSE53624 cohort, raw expression data were normalized and subjected to stringent quality control procedures. Genes with low expression levels (mean counts < 1 across all samples) were excluded. Differential expression analysis between ESCC tumor tissues and paired adjacent normal tissues was performed via limma 3.64.1 [15]. Genes with |log₂ fold change|>1 and *P* < 0.05 were significantly DEGs. The top 50 markedly up- and downregulated genes were presented via a heatmap.

### WGCNA and hub gene identification

WGCNA [16] was conducted to identify ESCC-associated gene co-expression modules (GCMs). The analysis was based on the top 5,000 most variably expressed genes in the GSE53624 cohort. Hierarchical clustering was applied to detect and remove outlier samples. The optimal soft-thresholding power was selected as the lowest value achieving a scale-free topology fit index (R²) greater than 0.8. GCMs were constructed using the dynamic tree-cutting method, with a minimum module size of 100 genes. Associations between modules and ESCC were evaluated by calculating Pearson correlation coefficients between module eigengenes and disease status. Modules with an absolute correlation coefficient |r|>0.7 and an adjusted P value < 0.05 were considered significant and selected for further analysis. Succinylation-related hub genes were identified by intersecting genes from these significant modules with differentially expressed genes (DEGs) and the predefined SRG set.

### Functional enrichment analysis (FEA) and PPI network construction

FEA of candidate genes was performed for Gene Ontology (GO) terms and Kyoto Encyclopedia of Genes and Genomes (KEGG) pathways via clusterProfiler in R 4.0.0 [17]. Enriched terms with a Benjamini-Hochberg false discovery rate (FDR)-adjusted P value < 0.05 were considered statistically significant. PPI networks and upstream transcriptional regulators were predicted using the STRING database (v11.5; https://string-db.org/), with a minimum interaction confidence score of 0.4.

### Prognostic signature development and validation

A prognostic signature was developed using the GSE53624 training cohort (*n* = 60). Feature selection was performed using least absolute shrinkage and selection operator (LASSO)-penalized Cox regression implemented in the glmnet package (R v4.1-10) with 5-fold cross-validation, enabling identification of genes with the strongest prognostic relevance from the candidate hub gene set [18]. A risk score model was constructed based on the optimal lambda value and subsequently applied to the internal test cohort (GSE53624, *n* = 59) and the external TCGA-ESCC cohort (*n* = 95) using the same gene coefficients. The optimal cut-off value distinguishing high- and low-risk groups was determined in the training cohort using the surv_cutpoint function (R v0.5.0), which is based on maximally selected rank statistics. This cut-off was consistently applied to all validation cohorts. Prognostic performance was evaluated using Kaplan-Meier survival curves in the training, internal test, and external validation cohorts, with group differences assessed by the log-rank test. Predictive accuracy for 1-, 3-, and 5-year overall survival (OS) was further evaluated using time-dependent receiver operating characteristic (tdROC) curve analysis.

### Nomogram construction

A multivariate Cox proportional hazards model was constructed to predict 1-, 3-, and 5-year OS. Clinicopathological variables with *P* < 0.05 in univariate analysis were incorporated into the multivariate model. A nomogram was subsequently generated using the rms package (v8.0-0). Model calibration was assessed using calibration curves based on 1,000 bootstrap resamples to evaluate agreement between predicted and observed outcomes.

### ICI analysis

The relative proportions of 22 immune cell types were inferred utilizing CIBERSORT (LM22 signature matrix; 1,000 permutations) [19]. Differences in ICI between high- and low-risk groups were assessed using the Kruskal-Wallis test. Spearman correlation analyses were performed to evaluate associations between the expression levels of the seven-gene prognostic signature and immune cell fractions, with results visualized as a heatmap. Single-sample gene set enrichment analysis (ssGSEA) was conducted based on 28 immune-related gene sets obtained from a previous study (https://www.cell.com/cell-reports/fulltext/S2211-1247(16)317090?_returnURL=https%3 A%2 F%2Flinkinghub.elsevier.com%2Fretrieve%2Fpii%2FS2211124716317090%3Fshowall%3Dtrue).

### miRNA-mRNA regulatory network construction

Following the prognostic signature establishment, an miRNA-mRNA regulatory network for the final signature genes was constructed. Putative miRNA-target interactions were identified by integrating predictions from multiMiR in R v3.0.0 [20] and miRTarBase (v9.0; https://mirtarbase.cuhk.edu.cn/).

### Drug sensitivity analysis

Drug sensitivity was estimated via pRRophetic in R [21] to estimate half-maximal inhibitory concentration (IC₅₀) values for anticancer agents. Predicted IC₅₀ values were compared between risk cohorts using the Wilcoxon rank-sum (WRS) test. Sensitivity scores were calculated based on transformed IC₅₀ values, with higher scores indicating increased drug sensitivity. A two-sided *P* < 0.05 suggested statistical significance.

### scRNA-seq validation

To validate the cellular localization of prognostic genes within the ESCC TME, scRNA-seq data from the ESCA_GSE160269 dataset were analyzed using TISCH (http://tisch.comp-genomics.org/).

### Statistical analysis

Analyses were enabled by R v4.5.1. Continuous variables were compared utilizing Student’s t-test for normally distributed data or the Kruskal-Wallis test for non-normally distributed ones. Two-sided *P* < 0.05 denotes statistical significance.

## Results

### Integrated transcriptomic and curated gene set analysis identifies a core set of SRGs in ESCC

**Fig. 1:**
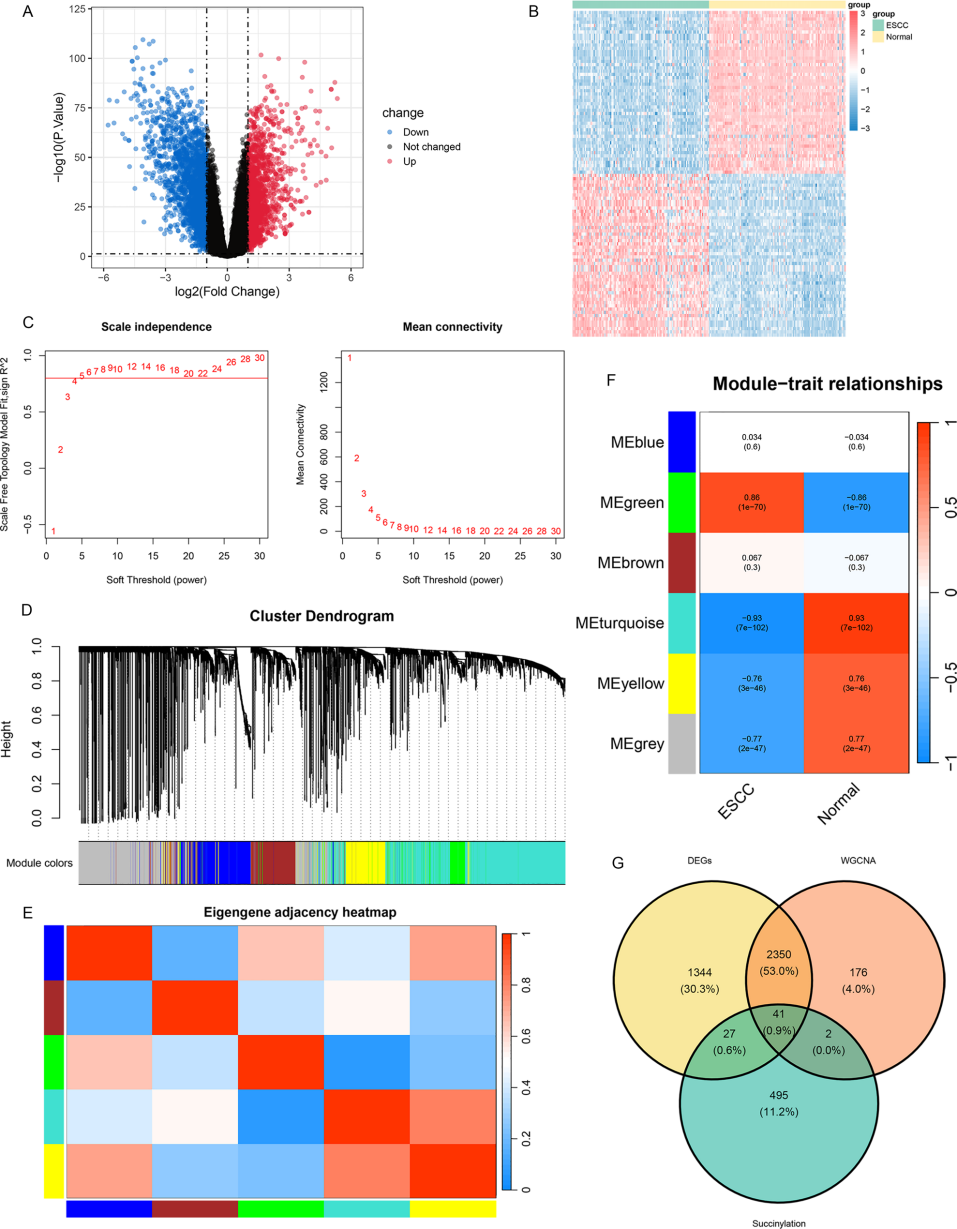
Integrated Multi-Omic Analysis Identifies SRGs in ESCC. **A** Volcano plot depicting 3,762 DEGs in ESCC in comparison to adjacent normal tissues. Red and blue dots indicate markedly upregulated (*n* = 1,581) and downregulated (*n* = 2,181) genes (|logFC|>1, *P* < 0.05); (**B**) Heatmap of the top 50 upregulated and downregulated DEGs, demonstrating clear separation between tumor (T) and normal (N) samples; (**C**) Network topology analysis across soft-thresholding powers (β). The selected power (β = 12) satisfies the scale-free topology criterion (R²>0.8); (**D**) Gene dendrogram clustered based on topological overlap, with module colors assigned correspondingly; (**E**) Heatmap illustrating correlations among identified co-expression modules; (**F**) Module-trait associations depicting correlations between module eigengenes and ESCC status; (**G**) Venn diagram displaying the intersection of DEGs, ESCC-associated module genes, as well as an SRG set, resulting in 41 high-confidence SRGs

Comprehensive transcriptomic analysis of the GSE53624 cohort identified 3,762 DEGs in ESCC tissues compared with adjacent normal tissues (|logFC|>1, *P* < 0.05), including 1,581 upregulated and 2,181 downregulated genes. The volcano plot showed a balanced distribution of up- and downregulated genes, indicating widespread transcriptional dysregulation associated with ESCC pathogenesis (Fig. 1A). A heatmap of the top 50 upregulated and downregulated DEGs clearly distinguished tumor from normal samples, supporting the robustness of the expression signature (Fig. 1B).

WGCNA was performed using the 5,000 most variably expressed genes. Network topology analysis identified β = 12 as the optimal soft-thresholding power for achieving a scale-free network topology (Fig. 1C). Genes were clustered into six co-expression modules (Fig. 1D), and inter-module correlations are presented in a heatmap (Fig. 1E). Module-trait relationship analysis revealed three modules significantly associated with ESCC status: the turquoise module (*r* = − 0.93, *P* < 0.001), the green module (*r* = 0.86, *P* < 0.001), and the yellow module (*r* = − 0.76, *P* < 0.001) (Fig. 1F).

Intersection analysis of genes from these three key modules, DEGs, and a predefined SRG set yielded 41 high-confidence SRGs (Fig. 1G).

### FEA and network analysis of succinylation-related hub genes

**Fig. 2:**
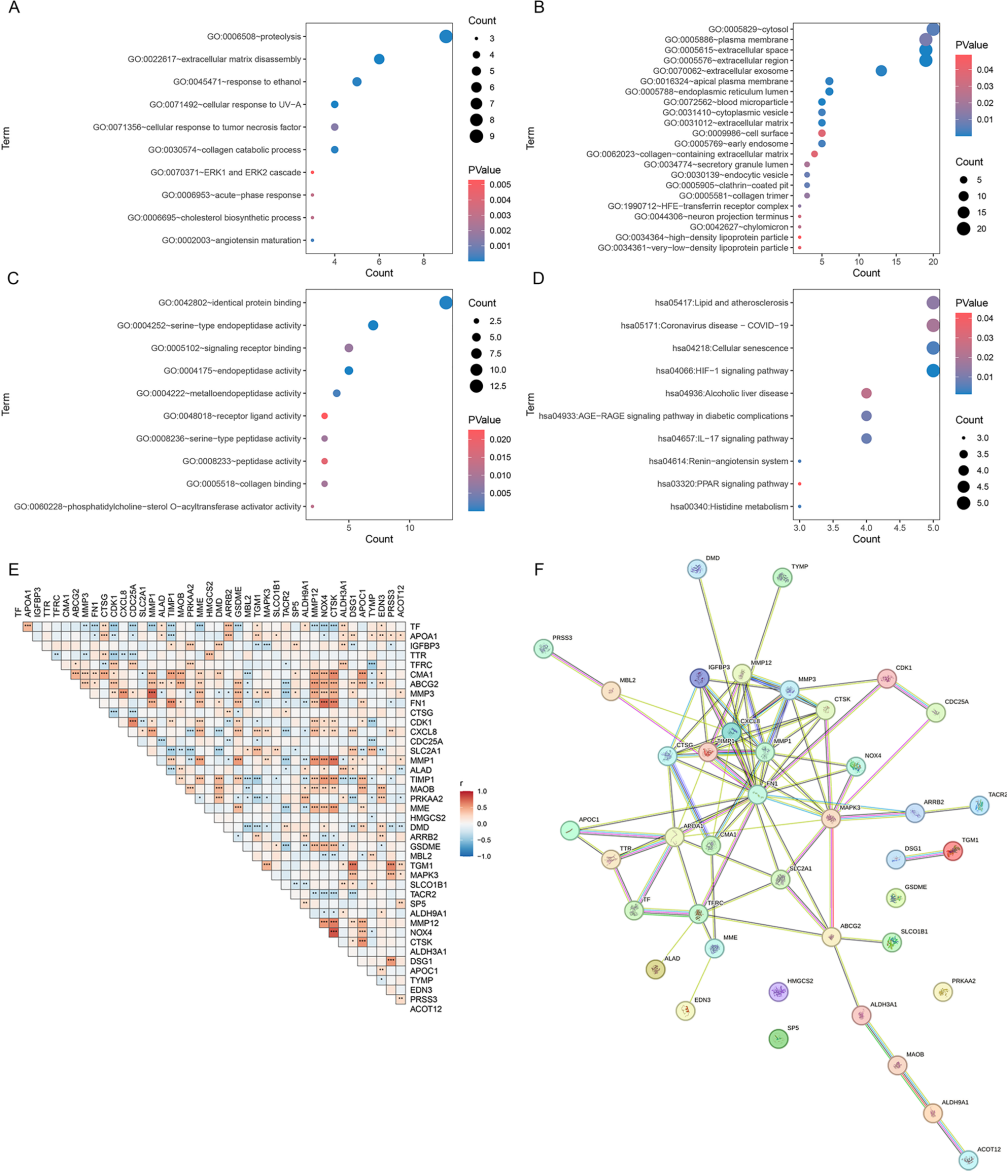
Integrated Functional Analysis of Succinylation-Related Hub Genes. **A** GO BP enrichment analysis, showing the top significantly enriched terms; (**B**) GO CC enrichment analysis, displaying markedly enriched CCs; (**C**) GO MF enrichment analysis, presenting key enriched MFs; (**D**) KEGG pathway enrichment analysis, showing notably enriched pathways; (**E**) Correlation heatmap of the 41 hub genes; red and blue indicate positive and negative correlation, respectively. Asterisks suggest statistical significance (*P < 0.05, **P < 0.01, ***P < 0.001); (**F**) PPI network of the hub genes

FEA of the 41 succinylation-related hub genes revealed their involvement in key biological processes (BPs). GO analysis demonstrated significant enrichment in extracellular matrix organization, proteolysis, and inflammatory response (BP, Fig. 2A). Cellular component (CC) analysis indicated predominant localization in the extracellular space, extracellular exosome, and extracellular region (Fig. 2B). Molecular Function (MF) analysis indicated enrichment in serine-type endopeptidase activity, metalloendopeptidase activity, and signaling receptor binding (Fig. 2C). KEGG pathway analysis [22] linked these genes to lipid and atherosclerosis pathways, cellular senescence, and IL-17 and AGE-RAGE signaling pathways in diabetic complications (Fig. 2D).

A heatmap illustrating expression patterns and pairwise correlations among the 41 SRGs revealed a complex co-regulatory network in ESCC (Fig. 2E). Numerous gene pairs exhibited strong positive correlations (red), while several showed significant negative correlations (blue), indicating intricate regulatory relationships among these genes.

PPI network analysis demonstrated dense connectivity among the hub genes (Fig. 2F), highlighting several highly interconnected nodes and supporting their potential cooperative roles in ESCC pathogenesis.

### Seven-gene prognostic signature development and validation

**Fig. 3:**
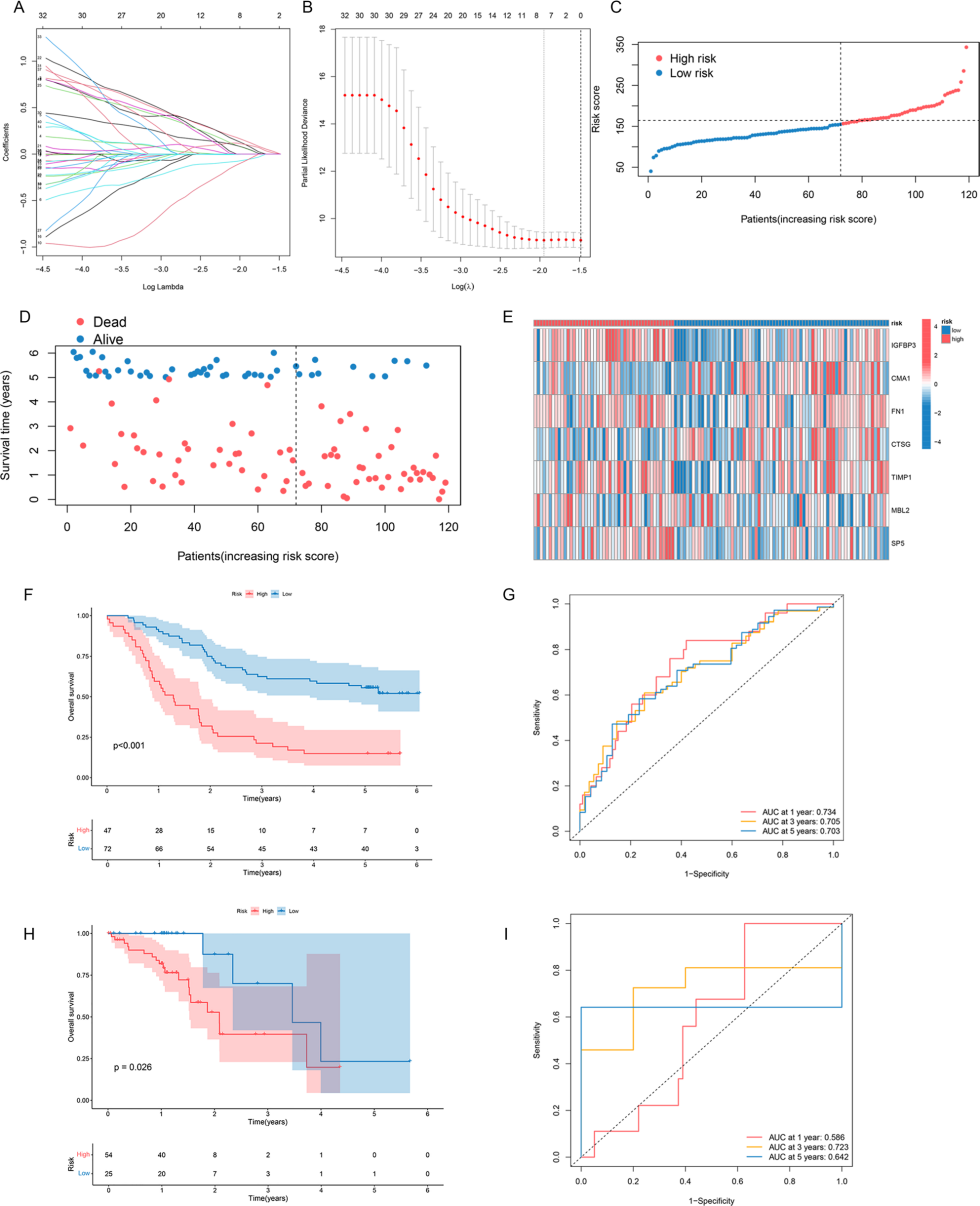
Seven-gene prognostic signature development and validation in ESCC. **A** Tuning parameter (lambda) selection in LASSO Cox regression; (**B**) Cross-validation curve (cvfit) of the LASSO regression; (**C**) Patient risk distribution in the training cohort. **D** Scatter plot depicting the relation of risk score to survival (Alive vs. Dead); (**E**) Heatmap illustrating expression patterns of the seven signature genes across patients in the training set; (**F**) KM survival curves for high- and low-risk cohorts in the GSE53624 training set (LRT, *P* < 0.0001); (**G**) tdROC curves evaluating predictive accuracy for 1-, 3-, and 5-year OS in the training set; (**H**) External validation via KM survival analysis in the independent TCGA-ESCC cohort (LRT, *P* = 0.026); (**I**) tdROC curves assessing predictive performance in the TCGA-ESCC cohort

A prognostic molecular signature for ESCC was constructed using the GSE53624 cohort through LASSO-penalized Cox regression analysis [23]. This analysis identified an optimal seven-gene prognostic signature comprising IGFBP3, CMA1, FN1, CTSG, TIMP1, MBL2, and SP5 (Fig. 3A, B). A risk score for every patient was computed via the prediction function based on expression levels.

Using the optimal cutoff value determined by surv_cutpoint, patients in the GSE53624 cohort were stratified into high-risk (*n* = 47) and low-risk (*n* = 72) groups. The distributions of risk scores, survival status, and expression profiles of the seven signature genes are shown in Fig. 3C-E, clearly differentiating the two risk groups. Kaplan-Meier analysis demonstrated significantly poorer OS in the high-risk group compared with the low-risk group (*P* < 0.001; Fig. 3F). Time-dependent ROC (tdROC) analysis further confirmed the strong predictive performance of the model, with AUCs of 0.734, 0.705, and 0.703 for 1-, 3-, and 5-year OS prediction, respectively (Fig. 3G).

Internal validation was performed by randomly dividing the GSE53624 cohort into training and validation sets at a 1:1 ratio. Consistent survival stratification and comparable predictive accuracy were observed in both subsets (Supplementary Fig. 1), supporting the robustness of the signature. External validation using the independent TCGA-ESCC cohort further demonstrated the generalizability of the model. Patients stratified into high- and low-risk groups showed significantly different survival outcomes (Fig. 3H, *P* = 0.026). tdROC analysis yielded AUCs of 0.586, 0.723, and 0.642 for 1-, 3-, and 5-year OS prediction, respectively (Fig. 3I). Collectively, this seven-gene signature represents a robust and generalizable independent prognostic indicator for ESCC patients.

### The seven-gene succinylation-based signature demonstrates robust prognostic value and clinical utility

**Fig. 4:**
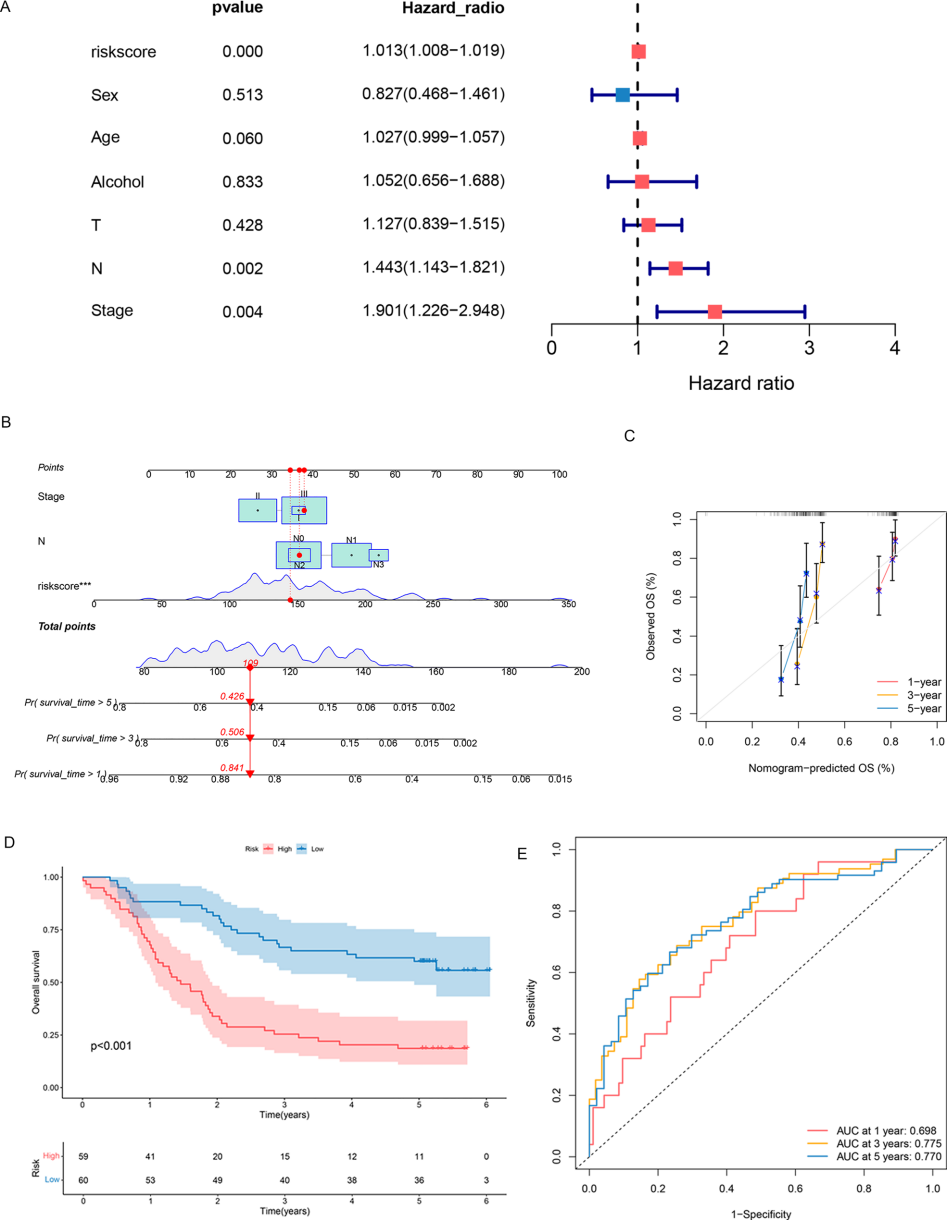
Prognostic independence and clinical utility of the seven-gene succinylation-related signature. **A** Forest plot of univariate Cox regression analyses for OS; (**B**) Prognostic nomogram integrating the risk score and clinical factors; (**C**) Calibration curves of the nomogram for 1-, 3-, and 5-year OS; (**D**) KM survival curves for high- and low-risk cohorts stratified by the signature; (**E**) tdROC curve analysis evaluating predictive accuracy for 1-, 3-, and 5-year OS

The independent prognostic value of the succinylation-based risk score was assessed alongside key clinicopathological variables, including age, sex, clinical stage, and alcohol consumption. Univariate Cox regression identified multiple factors significantly associated with OS (Fig. 4A). These significant variables were subsequently incorporated into a multivariate Cox regression model to construct a clinically applicable prognostic nomogram [24] (Fig. 4B). The nomogram enables quantitative estimation of 1-, 3-, and 5-year OS probabilities for individual patients. Calibration curves demonstrated excellent concordance between predicted and observed outcomes, confirming the accuracy and reliability of the model (Fig. 4C).

Patients were stratified into high- and low-risk groups according to the optimal cut-off derived from the total nomogram score. Kaplan-Meier analysis revealed significantly worse OS in the high-risk group (log-rank test, *P* < 0.001) (Fig. 4D). Predictive performance was further evaluated using time-dependent ROC analysis, yielding AUC values of 0.698, 0.775, and 0.770 for 1-, 3-, and 5-year OS, respectively. These results indicate superior prognostic accuracy compared with clinical staging alone (Fig. 4E).

In summary, these results solidify the clinical validity of our seven-gene succinylation-based signature. It serves not only as a potent independent prognostic biomarker but also as the core component of a highly accurate predictive tool, potentially guiding risk-adapted therapeutic strategies for the ESCC population.

### The succinylation signature reveals distinct immune microenvironment patterns in ESCC

**Fig. 5:**
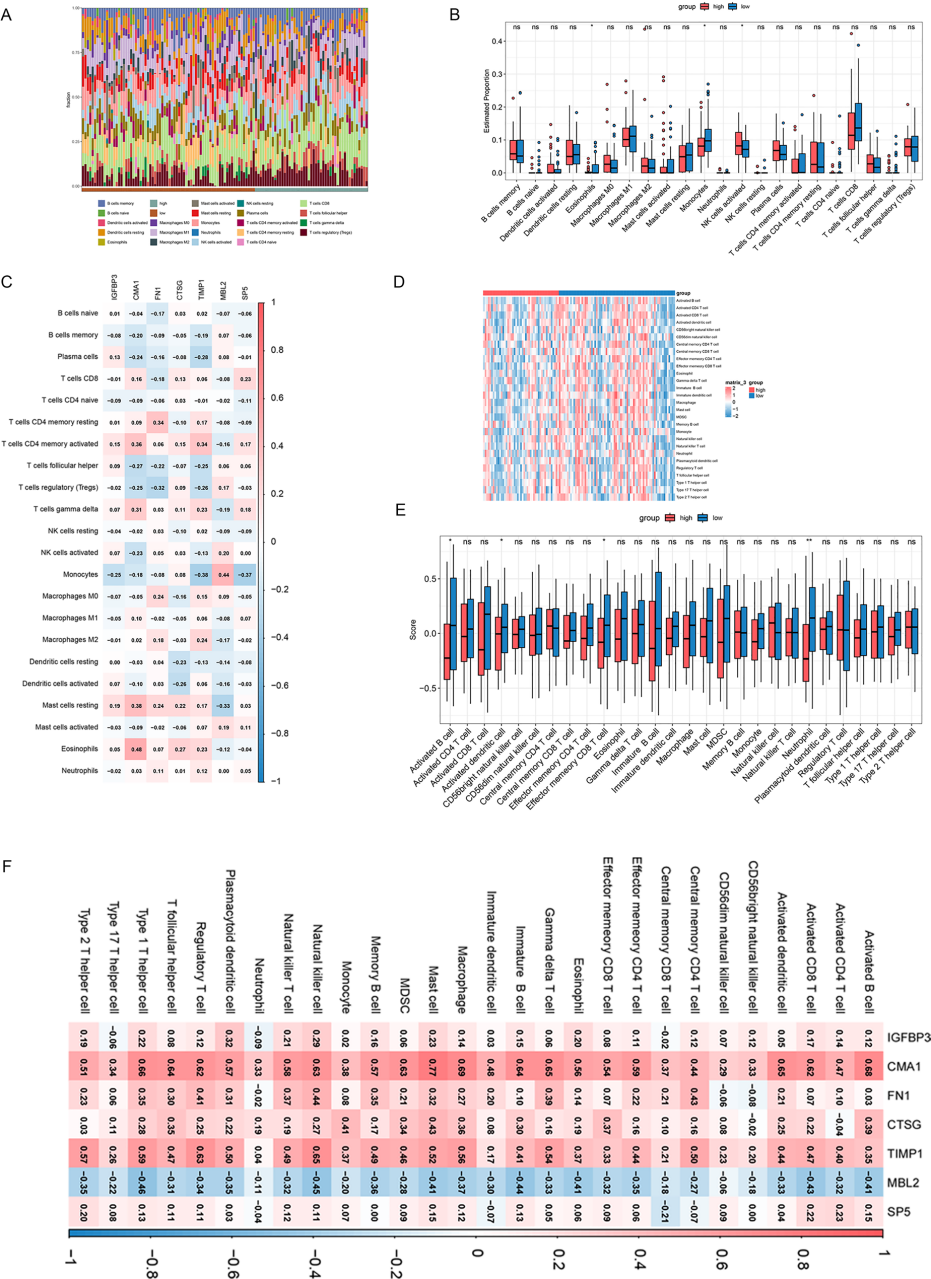
Comprehensive Immune Microenvironment Analysis Stratified by the Succinylation-related Risk Signature. **A** Stacked bar plot depicting the relative proportions of 22 immune cell types across all samples, as determined by CIBERSORT analysis; (**B**) Box plots comparing the infiltration levels of 22 immune cell types across high- and low-risk cohorts. Statistical significance was examined via the WRS test; (**C**) Heatmap of Spearman correlation coefficients between the seven signature genes and the infiltration levels of 22 immune cell types. Color intensity reflects the strength and direction of the correlations; (**D**) Heatmap illustrating ssGSEA enrichment scores for 28 immune-related gene sets across samples, annotated by risk group; (**E**) Box plots comparing ssGSEA scores of immune cell types across risk cohorts; (**F**) Correlation heatmap between the seven signature genes and ssGSEA scores of 28 immune cell types. *Statistical significance in panels (**B**) and (**E**) was determined via the WRS test (*P < 0.05; **P < 0.01; ***P < 0.001; ns, not significant). Color intensity in heatmaps (**C**, **D**, **F**) represents the magnitude and direction of correlation or enrichment

To elucidate the biological basis underlying prognostic stratification, the TIME was systematically characterized across risk groups. CIBERSORT analysis revealed heterogeneous infiltration patterns of 22 immune cell types among all samples (Fig. 5A). Comparative analysis identified significant differences in three immune cell populations: the high-risk group exhibited reduced infiltration of eosinophils (*P* < 0.05) and activated natural killer (NK) cells (*P* < 0.05), accompanied by increased monocyte infiltration (*P* < 0.05) (Fig. 5B).

Correlation analysis between the seven signature genes and ICI uncovered complex regulatory relationships (Fig. 5C). CMA1 showed a strong positive correlation with resting mast cells (*r* = 0.48), MBL2 was negatively correlated with monocytes (*r* = − 0.44), and FN1 demonstrated bidirectional associations, positively correlating with activated CD4 memory T cells (*r* = 0.34) while negatively correlating with regulatory T cells (*r* = − 0.32).

Consistent with these findings, ssGSEA based on 28 immune-related gene sets revealed clear separation between the two risk groups (Fig. 5D). Quantitative comparisons indicated significant attenuation of multiple antitumor immune pathways in the high-risk group (Fig. 5E). Correlation network analysis further highlighted MBL2 as a positive regulator of several immune populations, including activated CD8⁺ T cells, NK cells, and macrophages, whereas SP5 consistently showed negative associations with immune activation markers (Fig. 5F).

This comprehensive immune profiling confirms that the succinylation-derived risk signature stratifies ESCC patients into immunologically distinct subgroups, with the high-risk cohort featuring specific alterations in innate immune cell populations that possibly contribute to their adverse clinical outcomes.

### Regulatory network analysis and pharmacogenomic implications of the succinylation signature

**Fig. 6:**
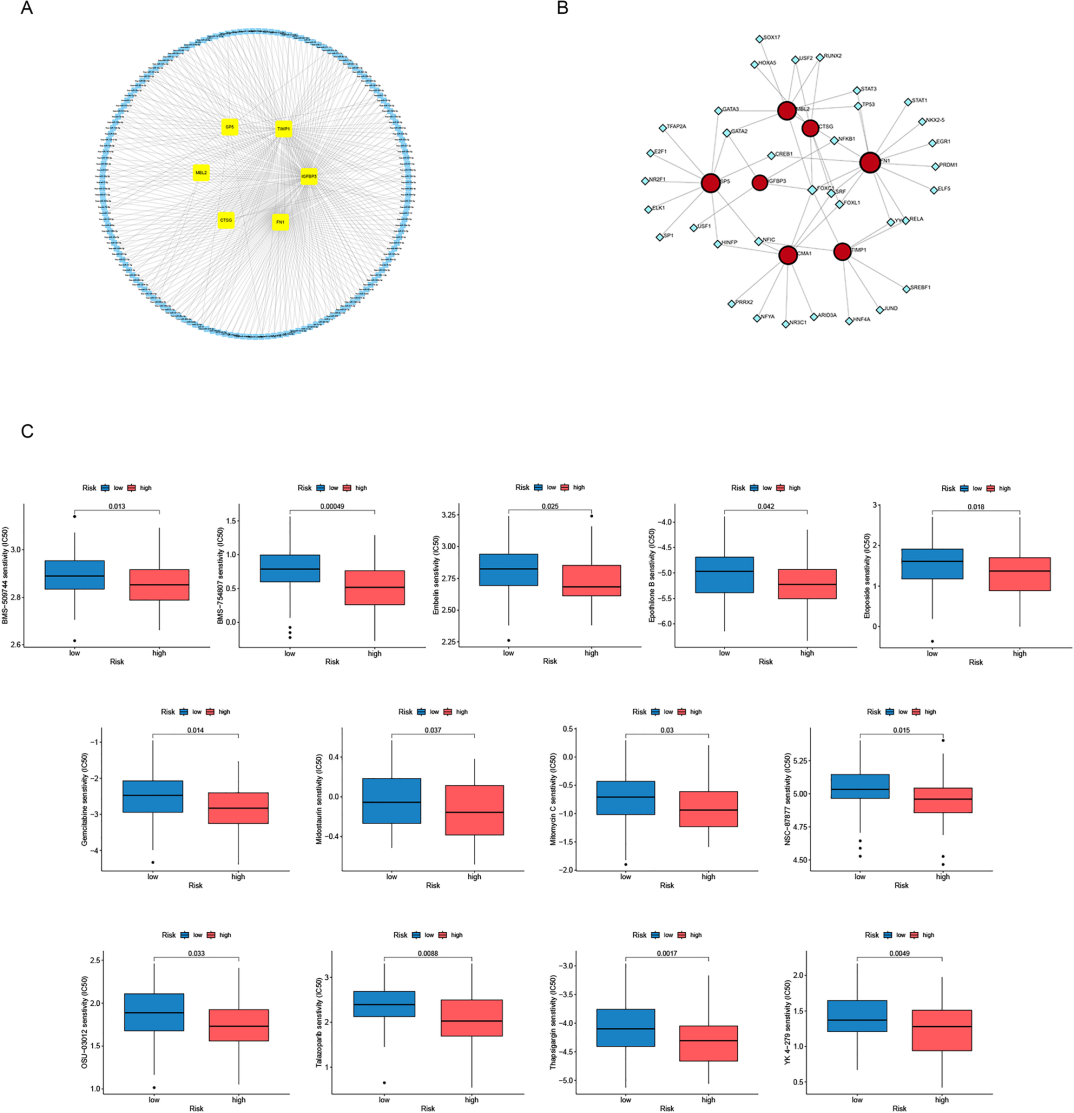
Regulatory Networks and Pharmacological Profiling of the seven-gene Succinylation Signature. **A** miRNA-mRNA regulatory network; (**B**) PPI network and upstream transcription factors, highlighting the core PPI network of the seven signature proteins; (**C**) Comparative drug sensitivity analysis. Box plots depict the distribution of sensitivity scores (transformed IC₅₀ values; higher scores indicate greater predicted sensitivity) for 13 statistically significant anticancer agents

To delineate the regulatory architecture and therapeutic relevance of the seven-gene succinylation signature, upstream miRNA regulators and protein-protein interactions (PPIs) were systematically analyzed. miRNA-mRNA network construction identified a panel of differentially expressed miRNAs predicted to target six signature genes (SP5, TIMP1, MBL2, IGFBP3, CTSG, and FN1) (Fig. 6A). PPI network analysis confirmed strong functional connectivity among the seven signature proteins and identified several potential upstream transcriptional regulators, including STAT3, RELA, and GATA3, suggesting a multilayered regulatory framework governing signature expression (Fig. 6B). In silico pharmacogenomic analysis revealed significant differences in predicted drug sensitivity between risk groups. Using the pRRophetic algorithm, IC₅₀ values were estimated for a panel of anticancer agents. The low-risk group exhibited significantly higher predicted sensitivity to 13 compounds compared with the high-risk group (Fig. 6C). These agents included kinase inhibitors (BMS-754807, Midostaurin), apoptosis inducers (Embelin, Thapsigargin), a PARP inhibitor (Talazoparib), and conventional chemotherapeutics with diverse mechanisms of action (Etoposide, Gemcitabine). Therefore, the high-risk succinylation signature is linked to a multidimensional resistance phenotype in ESCC, potentially influencing both targeted and chemotherapeutic efficacy, and providing a basis for future investigations into overcoming therapeutic resistance.

### Single-cell transcriptomic atlas and cellular specificity of signature genes in ESCC

**Fig. 7:**
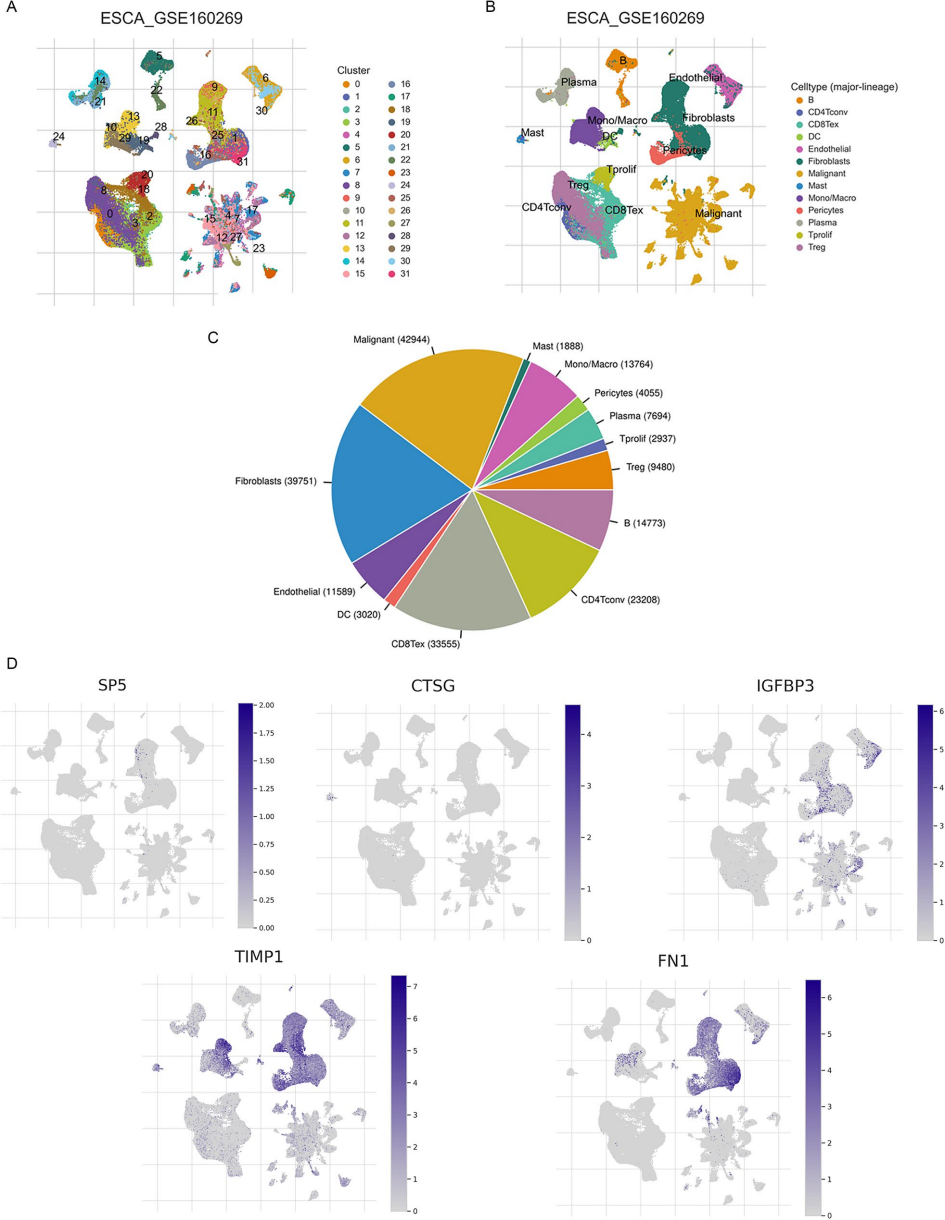
scRNA-seq Analysis of the ESCC TME and Cellular Localization of Signature Genes. **A **UMAP projection showing unsupervised clustering of all single cells from the GSE160269 dataset, colored by cluster number; (**B**) UMAP projection annotated with major cell types; (**C**) Pie chart depicting the proportional abundance of each major cell type within TME, with cell counts indicated; (**D**) Feature plots illustrating normalized expression levels of five succinylation-related signature genes (SP5, CTSG, IGFBP3, TIMP1, FN1) across the single-cell landscape

scRNA-seq (GSE160269) identified 32 distinct cellular clusters (Fig. 7A) and 13 major cell types annotated using canonical markers (Fig. 7B), including malignant cells, fibroblasts, endothelial cells, pericytes, dendritic cells, monocytes/macrophages, mast cells, B cells, plasma cells, and T-cell subsets (CD4Tconv, CD8Tex, Treg, and Tprolif). Malignant cells, fibroblasts, and exhausted CD8⁺ T cells constituted the predominant components of the TME (Fig. 7C). Cell-type-specific expression analysis of five succinylation-related prognostic genes revealed distinct spatial and cellular distribution patterns. SP5 was primarily expressed in malignant epithelial cells; FN1 and TIMP1 were co-expressed in malignant cells and cancer-associated fibroblasts; CTSG was enriched in myeloid populations; and IGFBP3 was predominantly expressed in fibroblasts (Fig. 7D). Therefore, the prognostic signature genes exhibit discrete cellular compartmentalization within the ESCC TME, suggesting roles in tumor-intrinsic malignant progression and immune-stromal interactions that possibly collectively influence patient outcomes. To move beyond cellular localization and better integrate the scRNA-seq findings with the prognostic model, fibroblast-centered cell-cell communication analysis was conducted using ligand-receptor interaction inference (Supplementary Fig. S2). Fibroblasts exhibited extensive inferred interactions with malignant and immune cell populations, suggesting that FN1/TIMP1- and IGFBP3-enriched fibroblast programs may function as key signaling hubs linking stromal remodeling with immune-tumor crosstalk, thereby providing a mechanistic context for the prognostic significance of the succinylation-based signature.

## Discussion

ESCC remains a highly lethal malignancy with a dismal prognosis and few reliable biomarkers [25]. The present study identifies a significant association between succinylation-related features and ESCC clinical outcomes. Using an integrative multi-omics framework, a novel seven-gene succinylation-related prognostic signature (IGFBP3, CMA1, FN1, CTSG, TIMP1, MBL2, and SP5) was developed and externally validated. This signature effectively stratifies patients into distinct risk groups independently of conventional clinicopathological parameters and characterizes an immunosuppressive TME that underlies the unfavorable prognosis observed in high-risk patients. Collectively, these findings implicate succinylation as an important regulator of ESCC progression and highlight its potential utility in risk stratification and tailored therapy. Notably, the term “succinylation-related” is operationally defined based on GeneCards and published literature; therefore, direct succinylation of these seven proteins, as well as their effects on their function or stability, cannot be inferred from the current data.

The prognostic performance of this signature is supported by the well-established, albeit context-dependent, roles of its constituent genes in cancer biology. FN1 and TIMP1 are closely associated with aggressive tumor behavior, consistent with previous reports. FN1 facilitates metastasis through extracellular matrix remodeling and activation of integrin-mediated signaling pathways [26, 27], whereas TIMP1, despite its name, frequently exerts oncogenic effects by promoting cell proliferation and chemoresistance [28–30]. In parallel, the immunosuppressive TME observed in high-risk patients, characterized by reduced NK cell infiltration and increased monocyte abundance, accords with findings across multiple cancer types [31, 32]. Attenuated NK cell infiltration compromises antitumor immune surveillance and is associated with adverse outcomes, while monocytes, often serving as precursors of tumor-promoting macrophages [33], contribute to the establishment of an immunosuppressive niche [34].

Importantly, this study provides PTM-oriented insights that highlight succinylation-related transcriptomic features associated with ESCC prognosis, underscoring the novelty of a PTM-centric approach. A notable example is IGFBP3: although some studies describe it as tumor-suppressive [35], high IGFBP3 expression in ESCC is consistently related to poor prognosis [36]. Nevertheless, direct experimental evidence demonstrating lysine succinylation-mediated regulation of IGFBP3 remains limited. Therefore, the present findings do not establish a definitive succinylation-dependent mechanism for IGFBP3 in ESCC. Any PTM-related functional interpretation of IGFBP3 should therefore be regarded as hypothetical and warrants targeted experimental validation. Furthermore, scRNA-seq revealed myeloid-restricted expression of CTSG, suggesting that the prognostic signal captured by the signature may, in part, reflect myeloid-associated transcriptional programs within the TME rather than a direct succinylation-mediated role of CTSG in eosinophil recruitment or differentiation [37, 38]. Notably, succinylation has been shown to modulate transcription factor activity in cancer. For instance, CPT1A-mediated succinylation of SP5 has been reported to enhance its transcriptional activity [39, 40].

From a conceptual perspective, these findings position succinylation as a potential metabolic-immune interface in ESCC. The inclusion of genes such as MBL2, a regulator of complement activation, and SP5, a key modulator of Wnt signaling, suggests that this metabolite-sensitive PTM may orchestrate crosstalk between tumor metabolism and immune evasion [41, 42]. Moreover, the distinct cellular localization of signature genes, malignant cells (SP5), cancer-associated fibroblasts (IGFBP3, FN1), and myeloid cells (CTSG), supports a model in which succinylation contributes to ESCC progression through coordinated cell-intrinsic and microenvironment-dependent mechanisms.

For clinical translation, the seven-gene signature and the accompanying nomogram may enhance risk stratification, enabling the identification of high-risk patients with early-stage disease who are more likely to benefit from intensified adjuvant therapy [43, 44]. Pharmacogenomic analyses further yield actionable insights: the predicted resistance to kinase inhibitors [45, 46] in high-risk patients suggests that combination strategies incorporating agents targeting protein succinylation merit exploration. In addition, the observed depletion of NK cells provides a rationale for investigating NK cell-engaging immunotherapeutic approaches in this subgroup [47, 48]. Moreover, the detection of circulating succinylated proteins, such as FN1 and TIMP1, through liquid biopsy represents a promising noninvasive strategy for dynamic disease monitoring [49].

There are also several limitations. The retrospective design restricts access to comprehensive treatment histories. Although the transcriptomic signature demonstrates prognostic value, an important limitation is that protein succinylation is inferred indirectly from mRNA expression rather than measured directly at the post-translational level. Notably, elevated mRNA expression does not necessarily correspond to increased succinylation of the encoded protein. This discrepancy likely reflects multiple regulatory layers between transcription and PTM, including post-transcriptional regulation, protein abundance and turnover, metabolic availability of succinyl-CoA, and the dynamic activities of succinyltransferases and desuccinylases that directly govern succinylation status. Furthermore, the operational definition of “SRGs” is based on GeneCards annotations and published literature, and no experimental validation has been performed to confirm whether the seven signature genes (IGFBP3, CMA1, FN1, CTSG, TIMP1, MBL2, and SP5) are succinylated in ESCC tissues or to quantify their succinylation abundance. Consequently, the proposed mechanistic link between the transcriptomic signature and succinylation-driven functional alterations, such as the role of succinylation as a key modifier of IGFBP3, remains speculative and lacks direct experimental evidence. Targeted validation is therefore required. For example, mass spectrometry-based proteomics could be employed to identify and quantify specific succinylation sites. In addition, while computational immune deconvolution is widely used, its limited spatial resolution constrains interpretation; spatial transcriptomics could provide more precise insights into interactions between succinylated protein-expressing cells and immune populations within the TME.

Future research should prioritize functional validation using site-directed mutagenesis in patient-derived organoids to establish causality, preclinical evaluation of succinylation-targeted therapies alone or in combination with immunotherapy, and prospective clinical studies to confirm the prognostic robustness and clinical utility of this model.

In conclusion, protein succinylation emerges as a master regulator of ESCC progression, integrating metabolic dysregulation, immunosuppression, and stromal crosstalk. The developed seven-gene signature provides a clinically actionable tool for risk stratification while revealing novel therapeutic vulnerabilities centered on PTM networks. These findings redefine therapeutic strategies for ESCC by targeting the succinylation-immunometabolism axis, providing a new roadmap for precision oncology.

## Supplementary Information

Supplementary Material 1.

Supplementary Material 2.

Supplementary Material 3.

## Data Availability

Data for this study were from the TCGA database ( https://portal.gdc.cancer.gov/ ) and the GEO database ( https://www.ncbi.nlm.nih.gov/geo/ ). Further inquiries can be directed to the corresponding author.
