# Supplementary figures and images for: Development and validation of a succinylation-related prognostic model for esophageal squamous cell carcinoma based on multi-omics bioinformatics analysis
Source: Hereditas. 2026 Feb 17;163:44. doi: 10.1186/s41065-026-00653-2 (PMC13015151; doi:10.1186/s41065-026-00653-2)

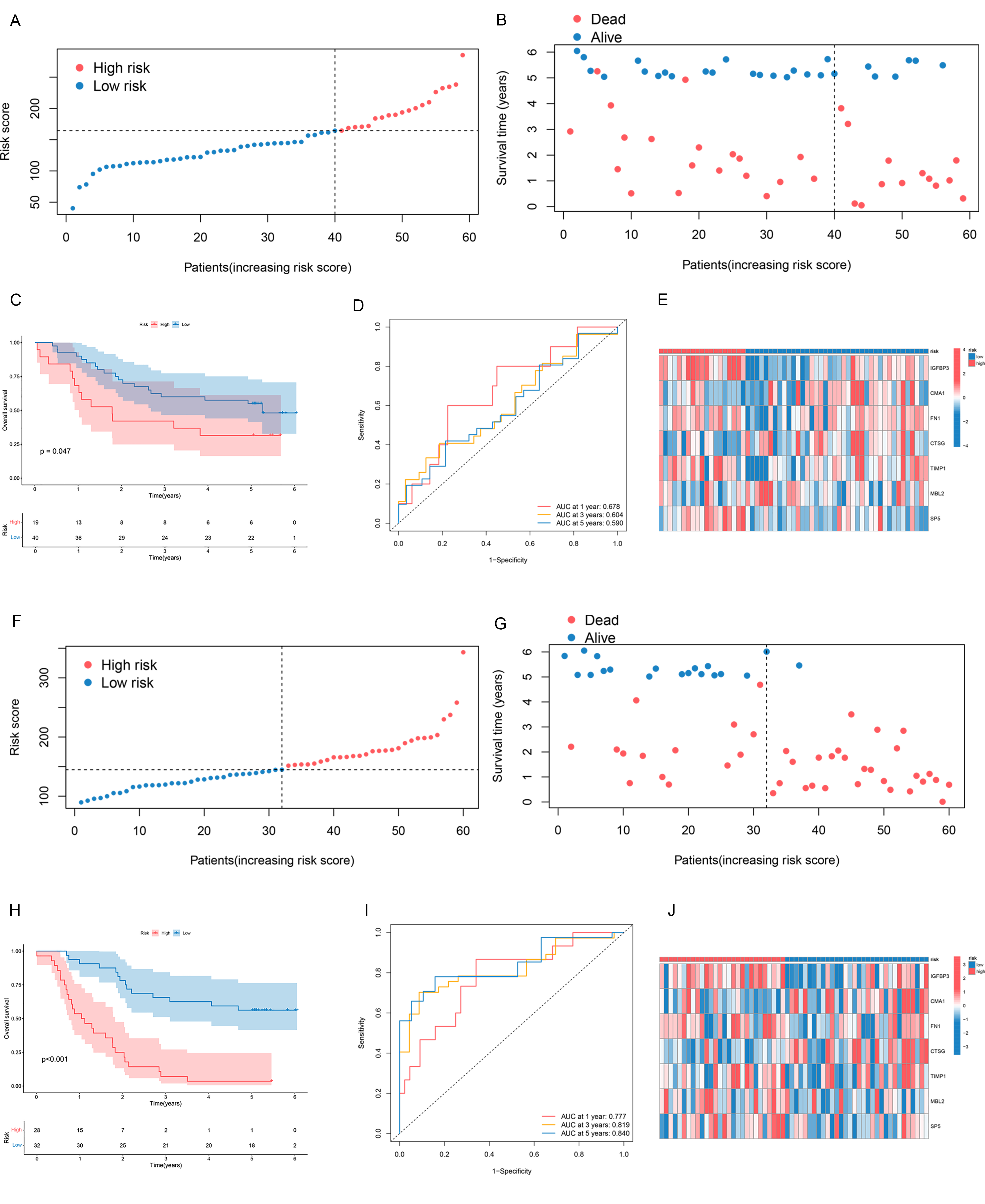

Supplement: Supplementary file 2 — Supplementary Material 2. [file 41065_2026_653_MOESM2_ESM.tif]

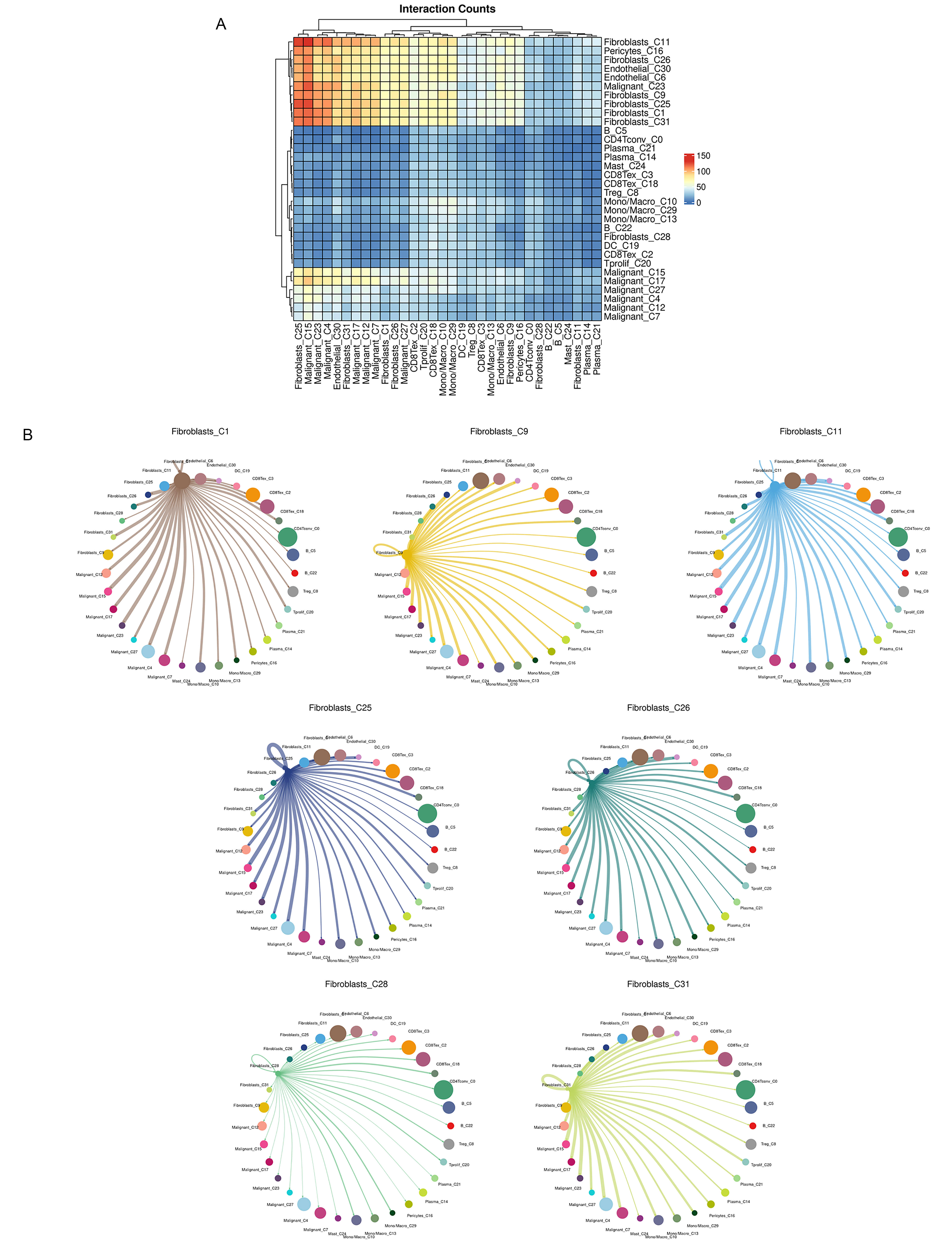

Supplement: Supplementary file 3 — Supplementary Material 3. [file 41065_2026_653_MOESM3_ESM.tif]
